# The ethical application of artificial intelligence in digital health: patients' knowledge, pulmonary hypertension, and the problem of misrecognition

**DOI:** 10.1093/ehjdh/ztag087

**Published:** 2026-06-15

**Authors:** Iain Armstrong, Peter Winter

**Affiliations:** Pulmonary Hypertension Association (PHA-UK), Thorncliffe park, Unit 1 Newton Business Centre, Newton Chambers Road, Chapeltown, Sheffield, S35 2PH and Pulmonary Vascular Disease Unit (PVDU), Royal Hallamshire Hospital, Glossop Road, Sheffield, S10 2JF; School of Sociology, Politics, and International Studies (SPAIS), University of Bristol, 11 Priory Road, Bristol, BS8 1TU, UK

**Keywords:** Patients’ knowledge, Pulmonary hypertension (PH), Misrecognition, Artificial intelligence, Co-design, Epistemic injustice

## Abstract

Artificial intelligence (AI) is increasingly being introduced into healthcare to improve efficiency, accuracy, and personalisation. Debate has centred on concerns such as bias, safety, and transparency. We argue that another problem deserves much more attention: misrecognition. By this we mean the risk that digital systems know patients mainly through what is easiest to measure and record, while overlooking what is hardest to code but most central to living with illness. Drawing on the concept of epistemic injustice, we suggest that patients may be disbelieved, misunderstood or required to translate complex, embodied, and relational experiences into clinical categories that fit poorly. Our point is not that patients' accounts fall outside data, but that all data require interpretation, and patient and caregiver inputs are often treated as lower-status knowledge in decisions about burden, benefit, and value. These risks do not arise uniformly across computational tools: they take different forms in task-specific machine learning systems used for classification or prediction, and in generative AI systems, including large language models, used to process or generate text. AI may deepen the problem by relying on proxies such as cost, utilization, and adherence thereby hardening narrow understandings of illness into technical systems. Using pulmonary hypertension (PH) as a case, we reflect on patient-led outcome measures such as emPHasis-10 to show that measurement is never neutral: it shapes what counts as legitimate knowledge, meaningful change, and good care. The key question is not only whether AI is accurate or fair, but what patient experiences become visible within it.

## Graphical Abstract

**Graphical Abstract:**
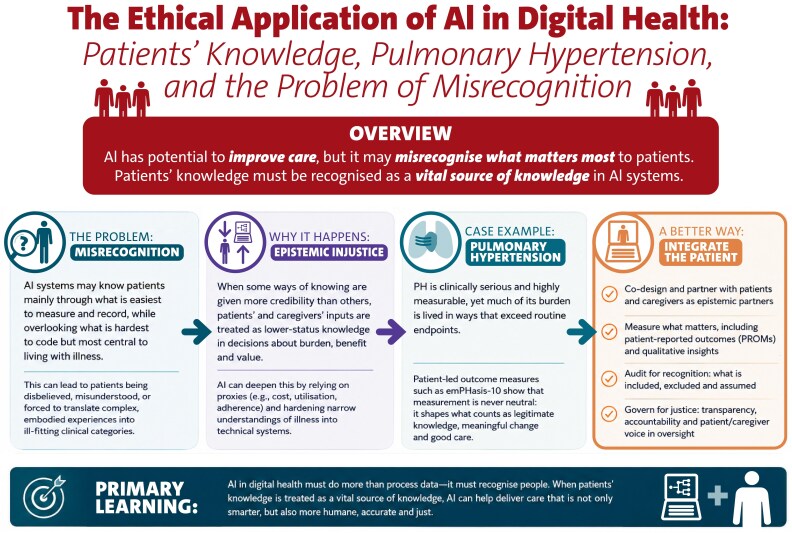

## Introduction

Artificial intelligence (AI) is increasingly presented as a route to more efficient, accurate, and personalized healthcare. Across digital cardiovascular health, AI is often linked to earlier diagnosis, better risk prediction, more streamlined workflows, and more responsive care.^1–3^ These ambitions are appealing, and in many cases, they are entirely reasonable. But there is another side to this story that still does not receive enough attention. In this Point of View, we use *digital health* in the World Health Organization sense, as the field of knowledge and practice associated with the development and use of digital technologies and data infrastructures to improve health.^4^ We use *AI* in the Organisation for Economic Co-operation and Development (OECD) sense, as machine-based systems that infer from the input they receive how to generate outputs such as predictions, recommendations, content, or decisions.^5^ Within that wider digital health infrastructure, AI is only one part of the picture, sitting alongside electronic health records, remote monitoring, patient portals, and other digital systems.

Artificial intelligence may come to know patients mainly through what is easiest to measure, standardize, and scale, while missing what is hardest to code but most important to living with illness. That matters because illness is not experienced only through physiological parameters, coded symptoms, or healthcare encounters. It is also lived through fatigue, fear, uncertainty, disrupted routines, altered relationships, and the constant work of adaptation. These dimensions are often central to how patients make sense of their condition, judge deterioration or improvement, and decide when and how to seek help. What is measurable and what is meaningful are not opposites. The problem, rather, is that health systems often privilege what is most readily measurable, even when that does not fully capture what matters most to patients.

This article begins from a simple claim: patients’ knowledge is not merely a source of data; it is a form of knowledge. Patients know things about illness that do not always sit neatly within routine datasets, scores, or categories. When that knowledge is sidelined, care can become not only less humane but less accurate and less just. We do not see a contradiction between data from patients and data from elsewhere. All data have to be interpreted, contextualized, and moved through what information science often describes as the data–information–knowledge–wisdom hierarchy.^6,7^ Our concern is that patients’ and, where relevant, caregivers’ inputs are too often treated as less relevant within that chain, even though they help determine what counts as deterioration, benefit, and value. To make sense of this problem, we draw on the concept of epistemic injustice and suggest that AI can act as a risk multiplier when healthcare systems already privilege what is measurable over what is meaningful.

We use pulmonary hypertension (PH) as a case example because it makes this tension especially visible. Pulmonary hypertension is clinically serious and highly measurable, yet much of its burden is lived in ways that can easily exceed what routine endpoints capture. This makes it a useful lens for thinking about digital health more broadly, especially when patient-reported outcomes are increasingly folded into AI-ready infrastructures. From there, we consider what this means for the design, evaluation, and governance of AI systems and argue that patients need to be treated not simply as data sources, but as epistemic partners.

## Why recognition matters

One helpful way of naming this problem comes from the idea of epistemic injustice. Fricker^8^ uses the term to describe a wrong done to someone specifically in their capacity as a knower. She distinguishes between testimonial injustice, where someone’s credibility is unfairly reduced, and hermeneutical injustice, where shared ways of understanding are too limited to make sense of someone’s experience.^8^ Put more simply, patients may not be believed, may be misunderstood, or may find that the language available in healthcare does not adequately capture what they are going through. Carel and Kidd^9^ and Kidd and Carel^10^ show why illness is such an important setting for this. Patients may be treated as less reliable because they are anxious, distressed, or seen as too subjective, and they may also struggle to make their experience intelligible within the language medicine gives them.^9,10^ In chronic conditions such as PH, caregivers may also hold important practical knowledge about day-to-day changes in symptoms, function, and treatment, as well as the burdens of digital care—e.g. the work of managing devices, tracking symptoms, responding to alerts, coordinating remote monitoring, and helping patients interpret or act on digital information—yet that knowledge is easy to overlook when formal systems privilege only clinical or administrative signals.^11^

This matters because the problem is not only interpersonal. It is also structural. Healthcare systems tend to favour what can be standardized, coded, compared, and audited. Artificial intelligence inherits these tendencies and can intensify them, because it often depends on structured variables, routinely collected datasets, and predefined endpoints.^1,2^ The ethical concern is therefore not that all AI tools are inherently biased. Rather, the risk is that some tools may misrecognize patients by relying on categories that fail to capture what matters most to them, while treating patients’ accounts as background context rather than knowledge in their own right. In digital health, this matters especially because systems that look neutral can quietly lock in narrow assumptions about what illness is, what counts as evidence, and whose account is taken seriously.

## Artificial intelligence as a risk multiplier

Artificial intelligence systems do not usually exclude patients altogether. More often, they include them in partial ways. They rely on variables such as utilization, adherence, coded symptoms, wearable outputs, and costs—i.e. service use, whether treatment appears to be taken as prescribed, symptom labels entered into records, information produced by wearable devices, and healthcare spending. These data can be useful, but they are also incomplete. They do not necessarily capture fear of deterioration, treatment burden, social isolation, the effort of self-management, or the everyday work patients do to understand their symptoms, notice changes, judge whether those changes are significant, and decide when to seek help. The same can be true for caregivers, who often help to monitor symptoms, manage appointments, support treatment routines, and interpret changes in the patient’s condition, but are not always treated as relevant end-users in the data and application cycle.^11,12^

This is not just a philosophical concern. It has clinical consequences. Obermeyer *et al*.^13^ showed that a widely used healthcare risk algorithm underestimated need among Black patients because it used healthcare cost as a proxy for illness severity. Since Black patients often incurred lower healthcare costs than White patients with similar needs, they had to be sicker to receive the same risk score. The wider lesson is clear: AI can reproduce inequity not only through biased data but through the proxies it uses to define need.

There is also the question of whether patients stay with digital systems at all. Greenhalgh *et al*.’s^14^ Non-adoption, Abandonment, Scale-Up, Spread, and Sustainability (NASSS) framework reminds us that technologies often fail not because they are technically weak but because they do not fit the realities of people’s lives. If patients stop using digital tools because the tools do not reflect what matters to them, that should not be dismissed as a simple implementation problem. It may be a sign that the system never really understood them in the first place.

## Pulmonary hypertension as a revealing case

Pulmonary hypertension offers a particularly clear example of this problem. It is measurable, serious, and clinically complex, but it is also lived through symptoms and burdens that can be hard to reduce to routine endpoints. Patients are often asked to translate breathlessness and fatigue into clinician-friendly categories, yet fatigue is not simply ‘tiredness’, and breathlessness is not only a physiological event. Both are also emotional, social, and existential experiences.

This is why PH is such a helpful case for thinking about digital health. It brings into view a familiar asymmetry: we tend to measure what is easy to measure and miss what is hard to live with.

Patient-reported outcome measures (PROMs) are one way of addressing this gap. They matter in their own right, not only because they may later become inputs to AI-enabled systems but because they represent an older and still unresolved debate about what healthcare chooses to measure and value. Reviews in PH have shown that the choice of PROM matters, because different instruments capture different domains and shape different understandings of what counts as improvement or deterioration.^15,16^ At the same time, PROMs should not be treated uncritically. They can capture symptoms, quality of life, and treatment burden from the patient’s perspective, but they may also be affected by reporting bias, expectations, placebo effects, and study design limitations; the appropriate response is not to marginalize PROMs, but to use them in well-designed, preferably alongside clinical, functional, or biological endpoints.^17^

The emPHasis-10 measure is especially useful here because it was developed from interviews with PH patients before psychometric validation.^18^ In other words, it started by listening. We are not arguing that emPHasis-10 is simply better than every other measure. Generic tools can be useful, especially when comparisons across conditions are needed. The particular value of emPHasis-10 is that it was built specifically around what people with PH said mattered, and it captures domains such as breathlessness, energy, control, confidence, independence, social restriction, and effects on family and friends.^15,18^ That PH-specific content is one reason disease-specific PROMs are often more sensitive to change in the aspects of life most disrupted by PH.^15,16^

That matters because it reminds us that measurement is not just technical—it is interpretive. It involves a judgement about what is worth counting and what is taken to matter. The implications of emPHasis-10 for AI design are summarized in *Table 1*.

**Table 1:** What emPHasis-10 teaches artificial intelligence designers about patients’ knowledge

Compared with generic tools, emPHasis-10 is brief, PH-specific, and built from patient-interviews about breathlessness, energy, confidence, independence, and effects on family life.^15,18^ PROM selection shapes what is treated as meaningful change in PH trials and practice.^15,16^ When outcomes foreground lived burden (e.g. daily functioning, independence), they challenge the assumption that physiological metrics alone represent ‘impact’.^15,16^ AI systems should not only collect data from patients but should also be shaped by what patients themselves identify as important outcomes and burdens.

The lesson is straightforward. Whatever we formalize as an outcome is what future systems are most likely to learn from, prioritize, and optimize. If AI is trained and judged mainly on clinically convenient endpoints, then even very sophisticated systems may end up reinforcing a thin account of what good care looks like. The aim, therefore, should not be to replace clinical endpoints with PROMs, but to build a stronger evidentiary base in which patient-defined value and clinical outcomes are assessed together.

## What metrics miss

*They asked how far I could walk. I told them how far I could hope.*

Clinical metrics give structure and comparability. They are indispensable. But they do not fully capture what it is like to live with breathlessness: waking in the night frightened you cannot draw air, the small victory of reaching the corner shop, or the humour people use to reframe what cannot be fixed. When AI systems are trained only on what is routinely recorded, they risk reproducing a narrow view of illness: the parts that fit into forms, codes, and dashboards.

This is not an argument against measurement. It is an argument for broadening what counts as evidence. Narrative medicine has long argued that good care depends on recognizing and interpreting patients’ stories, not only their scores.^19^ In digital health, work on free-text patient experience data suggests that unstructured narratives contain clinically relevant information that standard measures often miss and that natural language processing and machine learning may help organizations learn from these texts at scale, even if the methods remain variable.^20^

But simply adding narrative data does not solve the problem. If patients are not involved in deciding what questions matter, how stories are interpreted, and what follows from that interpretation, then narrative analytics can become just another extractive layer. The goal is not just richer data. It is epistemic partnership: patients having a say in the meaning and use of what is collected.

Blease^21^ adds another layer to this debate by noting that patients may disclose sensitive information more freely to AI tools than to clinicians. This concern is especially relevant to Generative AI and large language models, which invite conversational disclosure and can process or generate narrative text, rather than to every task-specific predictive model. Whether or not this becomes widespread, it raises an uncomfortable question: what are patients saying to digital systems that they do not feel able to say in clinical settings? If that is happening, then the problem is not only technological. It is also relational.

## Designing with patients, not simply around them

Responsible AI frameworks understandably stress privacy, transparency, accountability, and fairness.^22–25^ These are all important. But they can still leave a more basic question untouched: who gets to define the problem in the first place?

Co-design offers one response. Donia and Shaw^26^ argue that co-design matters in health AI, but also that it is difficult because machine learning systems change over time and can make it hard to see how user input affects eventual outcomes. For co-creation to be meaningful, it has to happen early, before outcomes, categories, and deployment goals become fixed. This applies to caregivers as well as patients, because caregivers often help to monitor symptoms, support treatment routines, manage appointments, use digital tools, and respond to alerts or advice. In chronic and rare conditions, they may therefore have important practical knowledge about whether a system is usable, burdensome, or clinically meaningful, yet digital health co-design reviews suggest that caregivers are still included inconsistently in development processes.^12^

The responsible innovation framework proposed by Stilgoe *et al*.^27^ is useful here because it asks innovators to stay open to challenges. Its four dimensions—anticipation, reflexivity, inclusion, and responsiveness—provide a way of thinking about digital health not as a fixed technical product, but as something that should be shaped and reshaped in conversation with those affected by it. Winter and Carusi^28^ illustrate this approach in PH by combining a professional foresight workshop with a patient focus group to identify and examine divergent expectations before they become embedded in technical plans.

There are also practical tools that can help. GRIPP2 and the UK Standards for Public Involvement offer ways of making patient involvement more transparent and robust.^29,30^ Reporting guidance such as Consolidated Standards of Reporting Trials - Artificial Intelligence (CONSORT-AI), Standard Protocol Items: Recommendations for Interventional Trials (SPIRIT-AI), Transparent Reporting of a multivariable Prediction model for Individual Prognosis or Diagnosis (TRIPOD + AI), and Prediction model Risk Of Bias ASsessment Tool for Artificial Intelligence (PROBAST + AI) can help make AI evidence clearer and easier to scrutinize.^31–34^ But none of these tools is enough if patients’ knowledge still comes in only after the main decisions have already been made. The practical implications of this argument are outlined in *Table 2*.

**Table 2:** Six commitments for digital health systems that better recognize what matters to patients and caregivers

Co-frame the problem with patients, and where relevant, caregivers, before ‘success’ becomes fixed in a specification.^12,26,28–30^ Select patient-defined outcomes, including PROM domains that reflect lived burden.^15,16,18^ Preserve narrative channels (free-text, diaries, open prompts) and treat patients’ and caregivers’ interpretation as shared work.^11,19,20^ Evaluate harms of misrecognition, such as labelling distress as ‘non-compliance’ or disengagement as ‘low motivation’.^14,20^ Interrogate proxies, especially costs and utilization, and test for inequitable impacts.^13,20^ Embed accountability and transparency using AI reporting and risk-of-bias guidance, alongside patient oversight.^31–34^

## Governance and incentives

Artificial intelligence in healthcare is shaped not only by technical capability but also by incentives, procurement systems, evaluation norms, and what can be demonstrated in trials or service settings. That means the risks are not only technical. They are institutional too. Bias and inequity can arise through data gaps, proxy measures, evaluation practices, and deployment contexts—problems that require governance as much as modelling.^23^

A minimal governance agenda for recognition-capable digital health would therefore include patient-defined value alongside clinical endpoints, transparency about intended use and trade-offs, post-deployment monitoring for inequitable impact and misrecognition, and stronger expectations around reporting and bias assessment when AI tools are introduced into care pathways.^31–34^ This is not an argument against AI. It is an argument for being clearer about what current evidence and current practice still leave unresolved.

## Conclusion

The debate around AI in digital health is not only about whether systems are accurate, fair, or efficient. It is also about whether they can recognize patients as knowers, rather than only as sources of data. Patient voice is not a soft extra. It is knowledge. When systems fail to recognize it, they do not simply lose nuance; they risk narrowing care itself. Epistemic injustice gives us a useful language for naming that harm, and for showing how digital systems can formalize and scale what counts as credible evidence in ways that matter clinically as well as ethically.^8–10^ The point is not that patients’ accounts cease to be data. It is that patient and caregiver inputs are too often treated as lower-status inputs in the chain by which data become evidence and evidence become decisions.^6,7^

Pulmonary hypertension is a helpful case because it makes a broader problem easier to see. The symptoms and burdens that matter most to patients are not always the ones most visible to systems, and not always the ones most heavily weighted in clinical and policy decisions. If AI is trained mainly on what is easiest to count, it may reproduce and amplify that mismatch. The result may be tools that are technically sophisticated, but still partial in what they are able to recognize.

That, we think, is the deeper challenge for digital health: not whether AI will arrive, but what it will be allowed to count as evidence, value, and good care. If patients are invited in only after these terms have already been set, we risk building systems that are efficient and impressive, but not fully responsive to lived illness. Recognition, in this sense, is not an optional extra added after technical development. It is part of what makes a system clinically meaningful in the first place.^13,14,26,31,34^

Just as importantly, we think these arguments should be taken back to patients themselves. Academic work on patients’ knowledge can easily reproduce the problem it seeks to expose if it speaks about patients rather than with them. For that reason, the next phase of this work will be developed through Pulmonary Hypertension Association UK's (PHA-UK) established patient networks, including patient advisory groups, member events, and the emPHAsis magazine community, to explore whether these arguments resonate with people living with PH, whether the harms described here reflect their experience, and what their responses might add to, revise, or challenge in our own thinking. We intend to begin that engagement during the next phase of this work, so that patient and caregiver perspectives can actively inform how this argument develops rather than being treated as a retrospective endorsement. Where daily management is shared, that conversation should include caregivers too, because their perspective can reveal practical burdens and forms of value that neither routine metrics nor system logs capture well.^11^ An article that argues for epistemic partnership should try to model it.

## Data Availability

No new data were generated or analysed for this Point of View article. The manuscript is based on conceptual analysis, published literature, and professional reflection; therefore, data availability is not applicable.

## References

[1] Lüscher TF, Wenzl FA, D’Ascenzo F, Friedman PA, Antoniades C. Artificial intelligence in cardiovascular medicine: clinical applications. Eur Heart J 2024;45:4291–4304. 39158472 10.1093/eurheartj/ehae465

[2] Armoundas AA, Narayan SM, Arnett DK, Spector-Bagdaddy K, Bennett DA, Celi LA, et al Use of artificial intelligence in improving outcomes in heart disease: a scientific statement from the American Heart Association. Circulation 2024;149:e1028–e1050. 38415358 10.1161/CIR.0000000000001201 PMC11042786

[3] Meder B, Asselbergs FW, Ashley E. Artificial intelligence to improve cardiovascular population health. Eur Heart J 2025;46:1907–1916. 40106837 10.1093/eurheartj/ehaf125 PMC12093147

[4] World Health Organization (WHO) . Global Strategy on Digital Health 2020-2027. Geneva: World Health Organization; 2025. https://www.who.int/docs/default-source/documents/gs4dhdaa2a9f352b0445bafbc79ca799dce4d.pdf.

[5] Organisation for Economic Co-operation and Development (OECD) . Explanatory memorandum on the updated OECD definition of an AI system, In: OECD Artificial Intelligence Papers No. 8. Paris: OECD; 2024. p. 1–11. https://www.oecd.org/content/dam/oecd/en/publications/reports/2024/03/explanatory-memorandum-on-the-updated-oecd-definition-of-an-ai-system_3c815e51/623da898-en.pdf

[6] Ackoff RL . From data to wisdom. J Appl Syst Anal 1989;16:3–9.

[7] Rowley J . The wisdom hierarchy: representations of the DIKW hierarchy. J Inf Sci 2007;33:163–180.

[8] Fricker M . Epistemic Injustice: Power and the Ethics of Knowing. Oxford: Oxford University Press; 2007.

[9] Carel H, Kidd IJ. Epistemic injustice in healthcare: a philosophical analysis. Med Health Care Philos 2014;17:529–540. 24740808 10.1007/s11019-014-9560-2

[10] Kidd IJ, Carel H. Epistemic injustice and illness. J Appl Philos 2017;34:172–190. 28303075 10.1111/japp.12172 PMC5324700

[11] Rawlings GH, Beail N, Condliffe R, Kiely DG, Thompson AAR, Sabroe I, et al Examining the impact of pulmonary hypertension on nonprofessional caregivers: a mixed-methods systematic review. Pulm Circ 2022;12:e12077. 35514773 10.1002/pul2.12077 PMC9063955

[12] Kilfoy A, Hsu TCC, Stockton-Powdrell C, Whelan P, Chu CH, Jibb L. An umbrella review on how digital health intervention co-design is conducted and described. Npj Digit Med 2024;7:374. 39715947 10.1038/s41746-024-01385-1 PMC11666587

[13] Obermeyer Z, Powers B, Vogeli C, Mullainathan S. Dissecting racial bias in an algorithm used to manage the health of populations. Science 2019;366:447–453. 31649194 10.1126/science.aax2342

[14] Greenhalgh T, Wherton J, Papoutsi C, Lynch J, Hughes G, A’Court C, et al Beyond adoption: a new framework for theorizing and evaluating nonadoption, abandonment, and challenges to the scale-up, spread, and sustainability of health and care technologies. J Med Internet Res 2017;19:e367. 29092808 10.2196/jmir.8775 PMC5688245

[15] Yarlas A, Mathai SC, Nathan SD, DuBrock HM, Morland K, Anderson N, et al Considerations when selecting patient-reported outcome measures for assessment of health-related quality of life in patients with pulmonary hypertension: a narrative review. Chest 2022;162:1163–1175. 35998707 10.1016/j.chest.2022.08.2206

[16] Varian F, Burney R, Pearson C, Goh ZM, Newman J, Rawlings G, et al Selection of patient-reported outcome measures in pulmonary arterial hypertension clinical trials: a systematic review, meta-analysis and health-related quality of life framework. Eur Respir Rev 2025;34:250006. 40368429 10.1183/16000617.0006-2025 PMC12076161

[17] Kluzek S, Dean B, Wartolowska KA. Patient-reported outcomes measures (PROMs) as proof of treatment efficacy. BMJ Evid Based Med 2021;27:153–155. 10.1136/bmjebm-2020-111573 PMC9132863 34088713

[18] Yorke J, Corris P, Gaine S, Gibbs JSR, Kiely DG, Harries C, et al emPHasis-10: development of a health-related quality of life measure in pulmonary hypertension. Eur Respir J 2014;43:1106–1113. 24232702 10.1183/09031936.00127113 PMC3971119

[19] Charon R . Narrative medicine: a model for empathy, reflection, profession, and trust. JAMA 2001;286:1897–1902. 11597295 10.1001/jama.286.15.1897

[20] Khanbhai M, Anyadi P, Symons J, Flott K, Darzi A, Mayer E. Applying natural language processing and machine learning techniques to patient experience feedback: a systematic review. BMJ Health Care Inform 2021;28:e100262. 10.1136/bmjhci-2020-100262 PMC7929894 33653690

[21] Blease C . Patients are disclosing sensitive information to AI tools—clinicians must adapt. BMJ 2026;392:s124. 41565342 10.1136/bmj.s124

[22] World Health Organization . Ethics and Governance of Artificial Intelligence for Health: WHO Guidance. Geneva: World Health Organization; 2021.

[23] Norori N, Hu Q, Aellen FM, Faraci FD, Tzovara A. Addressing bias in big data and AI for health care: a call for open science. Patterns (N Y) 2021;2:100347. 34693373 10.1016/j.patter.2021.100347 PMC8515002

[24] Azarfar G, Naimimohasses S, Rambhatla S, Komorowski M, Ferro D, Lewis PR, et al Responsible adoption of multimodal artificial intelligence in health care: promises and challenges. Lancet Digit Health 2025;7:100917. 41387134 10.1016/j.landig.2025.100917

[25] Alderman JE, Palmer J, Laws E, McCradden MD, Ordish J, Ghassemi M, et al Tackling algorithmic bias and promoting transparency in health datasets: the STANDING together consensus recommendations. Lancet Digit Health 2025;7:e64–e88. 39701919 10.1016/S2589-7500(24)00224-3 PMC11668905

[26] Donia J, Shaw JA. Co-design and ethical artificial intelligence for health. Big Data Soc 2021;8:1–12.

[27] Stilgoe J, Owen R, Macnaghten P. Developing a framework for responsible innovation. Res Policy 2013;42:1568–1580.

[28] Winter P, Carusi A. Professional expectations and patient expectations concerning the development of artificial intelligence (AI) for the early diagnosis of pulmonary hypertension (PH). J Responsible Technol 2022;12:100052. 10.1016/j.jrt.2022.100052 PMC9767405 36568032

[29] Staniszewska S, Simera I, Seers K, Mockford C, Goodlad S, Altman DG, et al GRIPP2 reporting checklists: tools to improve reporting of patient and public involvement in research. BMJ 2017;358:j3453. 28768629 10.1136/bmj.j3453 PMC5539518

[30] National Institute for Health and Care Research . UK standards for Public Involvement. London: NIHR; 2019.

[31] Liu X, Cruz Rivera S, Moher D, Calvert MJ, Denniston AK; SPIRIT-AI and CONSORT-AI Working Group. Reporting guidelines for clinical trial reports for interventions involving artificial intelligence: the CONSORT-AI extension. Nat Med 2020;26:1364–1374. 32908283 10.1038/s41591-020-1034-x PMC7598943

[32] Cruz Rivera S, Liu X, Chan AW, Denniston AK, Calvert MJ; SPIRIT-AI and CONSORT-AI Working Group. Guidelines for clinical trial protocols for interventions involving artificial intelligence: the SPIRIT-AI extension. Nat Med 2020;26:1351–1363. 32908284 10.1038/s41591-020-1037-7 PMC7598944

[33] Collins GS, Moons KGM, Dhiman P, Riley RD, Beam AL, Van Calster B, et al TRIPOD + AI statement: updated guidance for reporting clinical prediction models that use regression or machine learning methods. BMJ 2024;385:e078378. 38626948 10.1136/bmj-2023-078378 PMC11019967

[34] Moons KGM, Damen JAA, Kaul T, Dhiman P, Riley RD, Van Calster B, et al PROBAST + AI: an updated quality, risk of bias, and applicability assessment tool for prediction models using regression or artificial intelligence methods. BMJ 2025;388:e082505. 40127903 10.1136/bmj-2024-082505 PMC11931409

